# Collecting Duct Carcinoma of the Native Kidney in a Renal Transplant Recipient

**DOI:** 10.1155/2017/4527104

**Published:** 2017-09-14

**Authors:** Ian Zheng, Mahmoud Alameddine, Yaohong Tan, Zhobin Moghadamyeghaneh, Joshua S. Jue, Ali Yusufali, Ahmed Farag, Gaetano Ciancio

**Affiliations:** ^1^Department of Surgery, Division of Transplantation, Miami Transplant Institute, University of Miami Miller School of Medicine, Miami, FL 33136, USA; ^2^Department of Pathology, University of Miami Miller School of Medicine, Miami, FL 33136, USA; ^3^Department of Surgery, University of California, San Francisco, CA 94143, USA; ^4^Department of Surgery, Zagazig University School of Medicine, Zagazig, Egypt

## Abstract

Collecting duct carcinoma (CDC) is a rare and aggressive form of renal cell carcinoma (RCC) arising from the epithelium of Bellini's duct. It presents earlier in life and has a poorer prognosis than the clear-cell type. Historically, immunosuppressed renal transplant patients are more likely to develop malignancies than the general population. We report a case of CDC of the native kidney in a 59-year-old man who initially underwent kidney transplantation five years before the time of presentation. To our knowledge, CDC in the setting of renal transplant and long-term immunosuppression has not been previously described.

## 1. Introduction

Renal cell carcinoma (RCC) accounts for 2% to 3% of all adult malignancies [1]. The most common type of RCC is a clear cell, followed by papillary and chromophobe [2]. Collecting Duct Carcinoma (CDC) is a rare form of RCC that arises from the epithelium of Bellini ducts, located in the distal collecting duct of the renal medulla. It comprises about 1% of all RCC cases [3] and presents earlier in life and at a more advanced stage. CDC behaves more aggressively and is often unresponsive to conventional therapies [4]. Due to its rarity, definitive treatment for advanced and/or metastatic CDC has not been established.

In kidney transplant recipients, the estimated incidence of RCC is fivefold higher than in the general population [5]. An immunosuppressed status, deficient immune surveillance of malignant cells [6, 7], and reduced protection against oncogenic viruses [8, 9] may all contribute to the increased risk of cancer in transplant patients. The RCC subtypes of postrenal transplant patients include clear-cell carcinomas, chromophobe, and papillary carcinomas [10, 11]; however, CDC has not been previously reported in this setting.

The goal of this study is to report a novel case of postrenal transplant CDC in the native kidney that was complicated by distant metastases and to improve understanding of clinical and pathological characteristics. Treatment options for this disease in transplant patients will also be discussed.

## 2. Case Report

A 59-year-old male presented to the emergency department complaining of a 3-day history of suprapubic pain and macroscopic hematuria. The patient denied fever or dysuria. His physical examination was unremarkable, except suprapubic tenderness to palpation. Past medical history was consistent with smoking (30 pack-years, stopped 10 years prior to presentation), morbid obesity (135 Kg), hypertension for 7 years, diabetes mellitus for 15 years, hepatitis C virus (diagnosed during the pretransplant evaluation), and end-stage renal disease (on hemodialysis for 3 years). He received a kidney transplant from a deceased donor five years before the time of presentation. The donor was a 17-year-old male with no evidence of malignancy who died from a head injury. As part of the patient's pretransplant workup, an abdominal ultrasound revealed no kidney masses, but a liver biopsy showed stage 2 cirrhosis. The patient received induction immunosuppression both during and after the transplant that included thymoglobulin (1 mg/kg) and methyl prednisolone (500 mg Intravenous) for three daily doses and basiliximab (20 mg) for two daily doses. He was maintained on steroid-free immunosuppressant therapy consisting of low-dose tacrolimus (12-hour trough level: 6–8 ng/ml) and enteric coated mycophenolate sodium.

**Figure 1:**
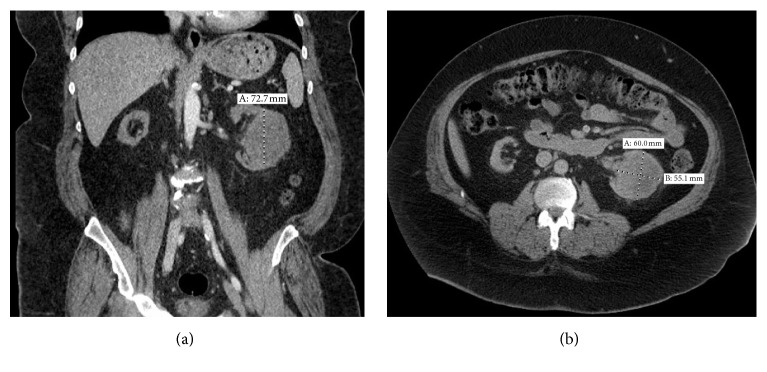
Coronal (a) and transverse (b) views of abdominal computed tomography demonstrate 7.27 × 6.00 × 5.50 cm heterogeneous mass in the left native kidney. Both kidneys show signs of atrophy.

In the emergency department, an abdominal ultrasound showed a normal appearing kidney allograft; however, the native kidneys were not properly visualized. For better assessment of the urinary tract, a computerized tomography scan (CT) was ordered. A suspicious heterogeneous mass was noted within the left native kidney that measured 6.0 × 5.5 × 7.3 cm in anteroposterior, transverse, and craniocaudal dimensions (Figure 1). Those findings were confirmed by MRI of the abdomen, raising the possibility of renal malignancy. No distant metastasis was identified. Consequently, hand-assisted laparoscopic radical nephrectomy was performed a few days later.

**Figure 2:**
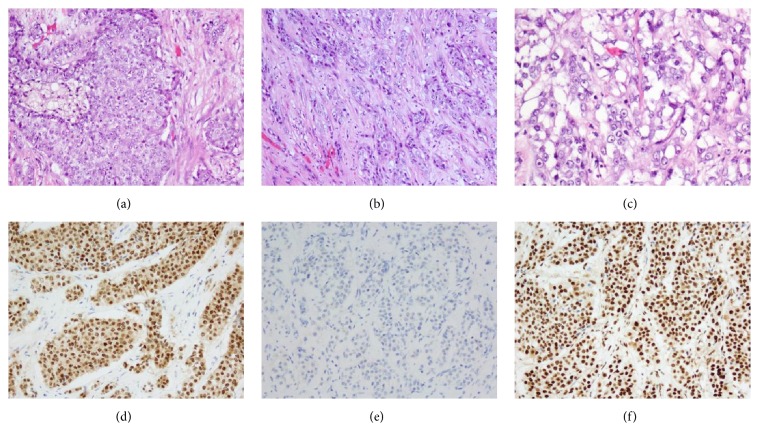
Histologic and immunophenotypic features of collecting duct carcinoma. (a) Tumor cells form solid sheets (20x). (b) Linear and single cell infiltration (20x). (c) Prominent large nucleoli (40x). (d) Positive for PAX8, consistent with renal origin. (e) Negative for GATA-3, ruled out urothelial origin. (f) INI-1 retained, ruled out medullary carcinoma.

Gross examination of the kidney revealed an ill-defined gray tan solid mass present in the middle lower pole of the left kidney, measuring 7.5 × 6.5 × 5.0 cm. The tumor extended into the renal sinus and perinephric adipose tissue with metastasis to a regional lymph node. Histologic examination demonstrated widely infiltrative high-grade adenocarcinoma with variable architectural patterns. Some areas showed solid tumor nests (Figure 2(a)), while another showed linear and single cell infiltration with prominent desmoplasia (Figure 2(b)). The tumor cells had marked nuclear pleomorphism, high nuclear : cytoplasmic (N : C) ratio, and prominent nucleoli (Figure 2(c)). Mitosis was brisk and the Fuhrman nuclear grade was 4 (ISUP/WHO Grade 4) [12]. The criteria for grade 4 include extreme nuclear pleomorphism and/or sarcomatoid and/or rhabdoid differentiation. In this case, sarcomatoid or rhabdoid component was not found; however, extreme nuclear pleomorphism was identified and characterized the specimen as a grade 4. Immunohistochemistry revealed that the tumor cells retained INI-1. The specimen was also positive for Pax8 and CK7 and negative for GATA3 and p63. Based on these morphologic and pathologic features, a diagnosis of collecting duct carcinoma was rendered. The American Joint Committee on Cancer (AJCC) pathology staging was pT3aN1M0.

The morphologic differential diagnosis included medullary carcinoma and urothelial carcinoma (UC). Medullary carcinoma is usually seen in patients with sickle cell trait, with a different history and patient age. Retention of INI-1 unequivocally ruled out medullary carcinoma. High-grade UC may also have similar morphology, although the absence of p63 and Gata-3 is inconsistent with high-grade UC.

Consequently, his immunosuppression regimen was modified to include sirolimus instead of mycophenolate sodium, as well as the addition of low-dose tacrolimus and oral prednisone. The patient was kept on close metastatic surveillance. Six months later, multiple bilateral pulmonary and bone metastases were noted on follow-up imaging. Cancer progression was confirmed pathologically by CT guided biopsy of the lesions. He was subsequently started on a tyrosine kinase inhibitor (TKI) in the form of sunitinib 37.5 mg daily for 4 weeks on and 2 weeks off as a first-line therapy while his immunosuppression regimen was adjusted to include tacrolimus and everolimus (0.75 mg twice daily). At that time, his kidney function was optimum (serum creatinine 0.6 mg/dl). Palliative radiation therapy was required to control his bone pain. Later, sunitinib was stopped after the development of drug toxicity, which included altered mental status, urinary tract infection, fatigue, anemia, leukopenia, thrombocytopenia, and diarrhea. However, the allograft function remained stable with a serum creatinine of 0.8 mg/dl. Follow-up imaging demonstrated worsening metastatic disease, so palliative care was established.

## 3. Description of Hand-Assisted Laparoscopic Radical Nephrectomy

After induction with general anesthesia and preparation of the surgical field, the patient was placed in a right semidecubitus position. Since the patient was morbidly obese with a huge truncal circumference, we performed the hand port at the left pararectal line (8 cm) to gain closer access to the left kidney. A Gelport with a 10 mm trocar was first placed into the abdominal cavity through the left pararectal incision. Under direct visualization, one 5 mm port was placed at the level of the ribcage at the midclavicular line, while the other was placed 3 cm superomedially to the iliac crest. The colon was initially reflected medially to the level of the aorta. The ureter was identified, released from its attachments, and divided. Using the electrothermal bipolar tissue sealing grasper, the upper pole was separated from the adrenal gland. In the approach to the renal hilum, the renal artery and vein were dissected and controlled by the vascular stapler. After the kidney and the tumor had been freed from surrounding attachments, the specimen was removed through the hand port. No complications were encountered, with an estimated blood loss of 200 mL and a total operative time of 3 hours.

## 4. Discussion

CDC is a rare pathologic type of RCC that behaves more aggressively and presents more often with metastatic disease than the clear-cell RCC. Early diagnosis is uncommon but most important to improve the prognosis of CDC patients. Diagnosis of CDC is often based on histopathological findings. The clinical symptoms of CDC are often nonspecific, including hematuria, flank pain, a palpable abdominal mass, or distant metastasis [13]. Typical CT findings include an inner medullary location, renal sinus involvement, infiltrative growth that can reach the cortex, heterogeneous and weak enhancement, and a cystic component [13]. Microscopic findings can be quite varied and may present with a variety of growth patterns, including tubular, papillary, microcystic papillary, pseudopapillary, cribriform, and solid patterns [14]. Nuclear atypia is often prominent and atypical mitotic figures are usually present [14]. In the center of the tumor, desmoplastic stromal reaction and infiltration with inflammatory cells can be observed. Histochemical analysis characteristically reveals low and high molecular weight cytokeratins,* Ulex europaeus *agglutinin 1, vimentin, and peanut lectin [14, 15].

Our patient presented with hematuria and CT findings of a large heterogenous mass that extended into the renal sinus, a common presentation for CDC. Microscopically, the patient's tumor showed features that ruled in CDC, including high-grade cytologic features with desmoplastic stroma and focal gland formation. A positive immunohistochemical stain for cytokeratins is characteristic for CDC and ultimately clinched the diagnosis.

The largest analysis of CDC addressing the survival and the demographic factors was done by Pepek et al. using the Surveillance, Epidemiology, and End Results (SEER) database. This study found that the 3-year relative survival rate for the localized, regional, and distant disease was 93%, 45%, and 6%, respectively [16]. Additionally, no significant difference was seen in disease presentation and relative survival based on race and sex [16].

Clinical research has been predominantly focused on clear-cell RCC rather than non-clear-cell due to the scarcity of non-clear-cell histology. In the majority of patients with CDC, surgery is the primary treatment. In more advanced cases, some studies have shown that patients respond to chemotherapy with gemcitabine and cisplatin (GC) [17, 18]. In a multicenter, prospective study that administered GC as first-line therapy, 23 CDC patients had a response rate of 26% and an overall survival of 10.5 months [19]. Targeted therapy, such as sunitinib, sorafenib, temsirolimus, and everolimus, has also been used with moderate success [20–22]. However, due to high rates of metastasis and local recurrence, nephrectomy and chemotherapy have not been found to effectively control aggressive forms of this disease [23]. About 2/3 of patients with CDC die within two years of its detection [14]. Since CDC frequently metastasizes and often presents with a poor prognosis, early detection and diagnosis are crucial.

Kidney transplant patients are at an increased risk of developing various types of malignancies. Renal transplant recipients are 3-4 times more likely to develop a malignancy, most notably integumentary and lymphoproliferative diseases, compared to patients on dialysis (transplant waiting list) [10]. Wong and Chapman also reported a small chance of transmission of cancer from the donors to the recipients [24]. This increased incidence of tumors also includes de novo malignancies, which are more aggressive and incur an increased mortality in transplant recipients compared to the general population [25]. For renal cancers, Kasiske et al. reported the incidence of RCC to be 15 times higher in the native kidney of patients after renal transplantation than in the general population and 39% higher than those on the kidney transplant waiting list [26]. According to Klatte and Marberger, the risk of RCC is increased in men, patients with acquired cystic kidney disease, African-Americans, recipients aged 65 years or older, donors aged 50 years or older, longer pretransplant dialysis time, and microscopic hematuria [27]. Renal cancer also occurs more often in the native kidneys (90%) than the graft kidney (10%) [28]. In another study, RCC of the native kidney in the context of a renal transplant was found to have an incidence within 0.35%–3.9%, with histologies consisting primarily of clear-cell carcinoma and papillary renal cell carcinoma [10]. Rare cancer subtypes of the native kidney postkidney transplant, such as collecting duct carcinoma, have not been described in the literature.

The increased incidence of malignancy in kidney transplant patients is complicated by the necessity of immunosuppressant therapy to prevent rejection of the renal allograft. Many studies have shown that prolonged immunosuppression plays a fundamental role in cancer occurrence. This is thought to do with impaired identification of tumors cells, diminished antiviral defense, and direct oncogenic effects of the drugs [29]. The typical maintenance immunosuppressive therapy for renal transplant recipients consists of a calcineurin inhibitor (CNI), an antimetabolite, and prednisone [30]. Growing evidence suggests that CNI (cyclosporine and tacrolimus) promote neoplastic changes due to the production of several cytokines that regulate tumor growth and metastasis [31].

A study by Javaid et al. indicated that sirolimus may be an alternative for both anticancer and antirejection effects [32]. Sirolimus is an orally administered inhibitor of mechanistic Target of Rapamycin (mTOR) that inhibits the production of interleukin-2 (IL-2). IL-2 is thought to enhance tumor regression and promote the immune response to neoplastic changes. The use of sirolimus has been associated with lower rates of squamous cell carcinoma of the skin and other malignancies in kidney transplant recipients [33, 34].

In the setting of RCC, everolimus, another inhibitor of mTOR, has shown better progression-free survival compared to placebo in patients whose disease had progressed on treatment with a TKI [35]. Since the duration and dose of immunosuppressant may be a contributing factor to the increased risks of malignancy, further studies are needed to find the optimal regimen for transplant recipients with malignancies. Our patient received a combined regimen consisting of sunitinib and everolimus. Nevertheless, drug toxicity from combining a TKI and mTOR inhibitor is a concerning issue. The results of a phase 1 study by Kanesvaran et al. [36] demonstrated that combination therapy with sunitinib and everolimus in the treatment of metastatic RCC was poorly tolerated. Despite lower everolimus doses used in kidney transplant patients, an exacerbated risk for drug toxicity still exists that adds to the deleterious effect of CNI. Several studies investigated the side effects of combining a TKI with an mTOR inhibitor. These trials used the combinations of sorafenib plus everolimus [37], sunitinib plus temsirolimus [38], and sunitinib plus everolimus [39]. Most of these studies were prematurely terminated due to significant toxicity that included thrombocytopenia, infection, hemorrhage, and gastrointestinal toxicity. Extra consideration should be given to the patient's renal function because of the deleterious effect of the targeted anticancer therapy on the kidney, especially in the setting of kidney transplantation [40].

Unfortunately, the prognosis for CDC patients is poor with all immunosuppressive agents. A previous study by Pickhardt found that less than 1/3 of patients survive more than two years [41]. This histology is commonly associated with metastases to the retroperitoneal lymph nodes, bones, lungs, and adrenal glands [20]. There is even a case with metastases to the heart, in a case report by Voss et al. [42], who recommended investigation with MRI in all patients with CDC and cardiac symptoms. Despite its typically aggressive behavior, cases without direct invasion or metastases have been described. In a case report by Hu et al., an early diagnosis of CDC was made after a presentation of right flank pain [23]. The tumor was locally resected, with no recurrence after ten months, suggesting that CDC may be very manageable if detected in an early stage. With posttransplant patients being at an increased risk of malignancy [10, 26], some authors have advocated for annual ultrasonography of native kidneys in transplant patients [11]. This may be especially beneficial in patients with congenital cystic disease, acquired cystic disease, or even a single cyst in native kidneys [28]. Further studies are needed to evaluate the risk of CDC with the use of immunosuppressive therapy.

In conclusion, CDC is a rare and aggressive disease that may present with gross hematuria. A full evaluation of the urinary tract, including the native kidneys, is essential in transplant patients presenting with gross hematuria. Modification of immunosuppression should be considered as part of the treatment for CDC. Routine evaluation of native kidneys for malignancy is advised for early detection of CDC in post-kidney-transplant patients. Further investigation is needed to determine the high-risk group for CDC patients who would benefit from the screening programs.
